# Rationalizing irrational prescribing—infection-related attitudes and practices across paediatric surgery specialties in a hospital in South India

**DOI:** 10.1093/jacamr/dlae105

**Published:** 2024-07-13

**Authors:** Surya Surendran, Vrinda Nampoothiri, Puneet Dhar, Alison Holmes, Sanjeev Singh, Esmita Charani

**Affiliations:** Department of Infection Control and Epidemiology, Amrita Institute of Medical Sciences, Amrita Vishwa Vidyapeetham University, Kochi, Kerala, India; Health System and Equity, The George Institute for Global Health, New Delhi, India; Department of Infection Control and Epidemiology, Amrita Institute of Medical Sciences, Amrita Vishwa Vidyapeetham University, Kochi, Kerala, India; Surgical Gastroenterology, Amrita Institute of Medical Sciences, Amrita Vishwa Vidyapeetham, Faridabad, India; Faculty of Health and Life Sciences, University of Liverpool, Liverpool, UK; National Institute for Health Research Health Protection Research Unit in Healthcare Associated Infections and Antimicrobial Resistance, Imperial College London, London, UK; Department of Medical Admin, Amrita Institute of Medical Sciences, Amrita Vishwa Vidyapeetham, Faridabad, India; Faculty of Health and Life Sciences, University of Liverpool, Liverpool, UK; Division of Infectious Diseases & HIV Medicine, Department of Medicine, Groote Schuur Hospital, University of Cape Town, Cape Town, South Africa

## Abstract

**Background and objectives:**

Antibiotic use in paediatric surgical specialties is understudied. We investigated the antibiotic prescribing practices of paediatric general and cardiovascular surgical teams in a tertiary hospital in South India.

**Methods:**

Mixed-methods study including observations from ward rounds, semi-structured interviews, and review of antibiotic prescribing. Field notes from observations and interview transcripts were coded using NVivo and thematically analysed. Data collection and analysis were iterative and continued until thematic saturation. Quantitative data were analysed using descriptive statistics.

**Results:**

Data included 62 h of observation, 24 interviews, one case study and 200 patient chart reviews (100/specialty). Senior surgeons make key decisions, referring to their own experience when prescribing antibiotics. Being outcome-driven, the doctors often prescribe antibiotics at the earliest indication of infection with a reluctance to de-escalate, even when an infection is not diagnosed. This practice is more acute among surgeons who consider themselves responsible for their patients’ health and attribute the consistently low surgical site infection rates to this practice.

In general surgery, 83.3% (80/96; 4 lost to follow-up) of patients were prescribed antibiotics for the duration of their stay with oral antibiotics prescribed at discharge. The surgeons use antibiotics prophylactically for patients who may be vulnerable to infection. The antimicrobial stewardship team was considered to have limited influence in the decision-making process.

**Conclusions:**

Outcome-driven decision-making in surgery leads to overprescription of antibiotics and prolonged surgical prophylaxis. The rationale for suboptimal practices is complicated by the surgeons’ beliefs about the contextual determinants of health in India.

## Introduction

Antimicrobial resistance (AMR) carries a disproportionate burden of negative outcomes in low- and middle-income countries (LMICs).^1–3^ Whilst international and national policies and interventions have been implemented to address AMR, including antimicrobial stewardship (AMS) programmes,^4–6^ varying impact has been reported depending on attitudes and behaviours across populations and differing specialties.^7,8^ The majority of efforts have focused on medical specialties, with limited activity in surgical specialties.^9^ Where interventions such as AMS have been implemented in surgery, their focus remains on surgical antibiotic prophylaxis and prevention of surgical site infection (SSI).^10,11^ Surgical antibiotic decision-making is prioritized differently, with emphasis on the surgical outcomes.^2,7^ This may lead to irrational and overuse of antibiotics in surgical specialties.^8^ The risk of antibiotic misuse is greater in paediatric populations due to lack of data on antibiotic dosing in this population, lack of effective paediatric stewardship programmes and inability for effective diagnostics and culture and susceptibility testing in this population.^12–14^

To date, there have been few studies focusing on management of infections and AMS in inpatient paediatric populations, particularly in LMICs.^15^ A systematic review, on implementation of AMS in paediatrics, identified more than half the included studies were from the USA, with 18% (20/113) studies from Asia.^16^ The studies in LMICs focus on surgical prophylaxis,^17–20^ overlooking the extent of post-operative empirical antibiotic use. A recent study, reporting on survey findings conducted with participants from over 66 countries indicated that LMICs have a lower number of infection prevention and control (IPC) and AMS programmes compared with high-income countries.^21^ Very few studies have looked beyond the numbers and explored the contextual determinants of antibiotic prescribing in paediatric inpatient settings in LMICs.

In this study, we investigated the antibiotic prescribing behaviours and attitudes across two paediatric surgical specialties in a tertiary hospital in South India.

## Methods

### Study setting

This study was conducted in the paediatric general surgery (PSx) and paediatric cardiovascular thoracic surgery (PCVTS) at a 1350-bed tertiary surgical referral teaching hospital in India with various adult and paediatric specialities. The PSx team has expertise in gastrointestinal, hepatobiliary and urology surgery, among others, while the PCVTS team performs a wide range of cardiovascular surgery including heart valve repair, neonatal heart surgery and complex biventricular conversion, to name a few. The hospital has a clinician-led, pharmacist-driven AMS programme.^5,22^

Ethical approval for this study was granted by Amrita Hospital Institutional Research and Ethics Committee (IRB-AIMS-2019-001).

### Data collection

Between March 2019 and September 2019, researchers conducted an ethnographic study involving non-participant observations, interviews and documentary analyses using a data collection guide, developed through review of literature and previous work of the research team.^2,7^ Ethnographic observations, together with documentary analyses and face-to-face interviews, provided insight into the key processes in the surgical pathway in relation to infection-related care from both the healthcare-provider and the healthcare-user perspectives.

### Documentary analysis

Data were collected from medical records of admitted patients of both specialties for whom empirical antibiotics other than for prophylaxis were prescribed. Specially designed data collection forms were used for this purpose (File S1, available as Supplementary data at *JAC-AMR* Online). Data collected included patient demographics, details of antibiotic prescribed, along with inflammatory markers and microbiology reports of culture and susceptibility, if available. Indication for antibiotics was taken from the progress reports written by the treating doctor, when available. Antibiotics prescribed were categorized as per the WHO access, watch and reserve (AWaRe) classification.^23^

### Ethnographic observations

Two researchers followed the surgical team members across the pathway from outpatient (OP) clinic to ICU and inpatient wards. Observations were conducted using a pre-piloted data collection guide that provided information to be collected for each episode of observation, ensuring that the notes were consistent in detail and content. Prior to data collection, the participants were informed about the study and informed consent was obtained. One patient with a prolonged hospital stay due to an infection was identified during ward rounds for an in-depth case study.

### Face-to-face interviews

Following the observations, healthcare workers involved in the patient pathway were invited for interview. The interviews occurred on site at mutually agreed locations, away from clinical duties at a time convenient to the participants. Written consent was obtained from the participants prior to the interviews. A semi-structured interview guide, developed through review of literature and previous work of the research team, was used. The interviews were also an opportunity to clarify queries or scenarios the researchers had from the focused observations. The interviews were audio-recorded and transcribed verbatim. The interviews of nurses were conducted in the local Malayalam dialect. These interviews were transliterated to English, and then cross-checked for accuracy. All other interviews were conducted in English.

### Case study

The case study was generated by in-depth documentary analysis (patient medical notes, medication charts, clinical results) using a piloted template that captured the key relevant episodes of care from each stage of the pathway for individual patients after obtaining consent from the carer. Each documented episode of care was carefully scrutinized and data on all variables (e.g. microbiology results, antibiotics prescribed, medical history, surgical interventions) were collected. The healthcare professional, carer and patient narratives and experiences were also captured through focused interviews.

### Data analysis

Field notes and interview transcripts were analysed thematically, aided by NVivo software. The fieldnotes and interview transcripts were coded line by line and multiple discussions held among the three-member team resulted in finalizing the codebook and thematic structures. Analysis and data collection were iterative and recursive, using constant comparison. Descriptive statistics were used to analyse the quantitative data using Microsoft Excel.

## Results

Data included 62 h of observation (27 h in PCVTS, 35 h in PSx), 24 interviews (11 in PCVTS, 5 in PSx, 8 staff nurses), and 1 case study (PSx). The staff nurses catered for both departments on a rotational basis; therefore, the presented value encompasses data from both departments. Antibiotic prescriptions of 200 patients were reviewed (100 from each specialty).

### Description of quantitative data collected

We collected complete data for 96 patients from PSx (4 lost to follow-up) and for 98 patients from PCVTS (2 lost to follow-up) during the study period (Table 1).

**Table 1.** Demographics and key antibiotic related data collected

	Demographics and key antibiotic-related data collected	PSx, *n* (%)	PCVTS, *n* (%)
Total		96 (100)	98 (100)
Age^a^	Neonates (0–27 days)	15 (15.6)	7 (7.14)
	Infants (28 days–12 months)	35 (36.4)	53 (54.1)
	Toddlers (13 months–2 years)	13 (13.5)	15 (15.3)
	Early childhood (3–5 years)	18 (18.8)	11 (11.2)
	Middle childhood (6–11 years)	9 (9.4)	7 (7.2)
	Early adolescents (12–18 years)	6 (6.25)	5 (5.1)
Sex	Female	32 (33.3)	36 (36.7)
	Male	64 (66.7)	62 (63.3)
Length of hospital stay, days	<5	21 (21.9)	0 (0)
	5–10	16 (16.7	7 (7.1)
	10–15	34 (35.4)	14 (14.3)
	15–20	10 (10.4)	26 (26.5)
	20–25	4 (4.2)	11 (11.2
	≥25	11 (11.5)	40 (40.8)
	Average	12.5	26
	Median	11	20.5
Culture positivity	Blood	4 (4.2)	15 (15.3)
	Surgical site	11 (11.5)	6 (6.1)
	Respiratory	4 (4.2)	15 (15.3)
	Urinary	7 (7.3)	4 (4.1)
	More than one type of positive culture	3 (3.1)	10 (10.2)
Antibiotics^b^	PSx		
	Patients with routine antibiotics	68 (70.8)	NA
	Antibiotics prescribed with positive culture	8/68 (11.8)	NA
	Antibiotics prescribed without positive culture	60/68 (88.2)	NA
	Antibiotics other than the regular regimen	28 (29.1)	NA
	Antibiotics prescribed with positive culture	12/28 (42.9)	NA
	Antibiotics prescribed without positive culture	16/28 (57.1)	NA
	PCVTS		
	Antibiotics prescribed with positive culture	NA	30 (30.6)
	Antibiotics prescribed without positive culture	NA	68 (69.4)
Infections markers at time of antibiotic commencement	Antibiotics given when temp ≥37°C	4 (4.2)	41 (41.8)
	Patients with WBC results before antibiotic prescription	59 (61.5)	76 (77.6)
	Abnormal WBC count^c^	25/59 (42.4)	13/76 (17.1)
	Patients with platelet results before antibiotic prescription	49 (51)	74 (75.5)
	Abnormal platelet count^d^	16/49 (32.7)	29/74 (39.2)
Antibiotics on discharge	Yes	80 (83)	15(15)

^a^Age was categorized using the National Institute of Child Health and Human Development (NICHD).^24^

^b^Since PSx had a regular regimen for all patients admitted to the department, we separated out the culture data for those who were prescribed only the regular regimen and those who were prescribed antibiotics in addition to the regular regimen.

^c^Normal range = 6–16  × 10^3^/μL.

^d^Normal range = 200–550 × 10^3^/μL.

The indication for starting antibiotics was not consistently provided by prescribers. The most common documented indication for which antibiotics were prescribed in PCVTS was fever, followed by rise in C-reactive protein (CRP), reduced WBC count and/or platelets. In PCVTS, 69/98 cases where there was no positive microbiological culture had an antibiotic prescribed. Of these, 31.8% (22/69) had a temperature spike (one reading of body temperature ≥37°C). Of the patients for whom an antibiotic was prescribed in PCVTS, 17.1% (13/76) and 39.2% (29/74) had an abnormal WBC and platelet count, respectively, out of the total recorded WBC (76/98) and platelet counts (74/98). As per the AWaRe classification, watch category antibiotics were the most prescribed prescriptions (79%, 77/98). In PSx, routine practice included antibiotic regimens to be started as prophylaxis (usually started the day before surgery) and for it to continue post-operatively, with no documentation of indication. The regimen included a combination of three antibiotics: cefotaxime, amikacin or ofloxacin, and metronidazole. This combination was prescribed in 70.8% (68/96) of patients upon admission; of these patients, 88.2% (60/68) had the combination therapy started with no positive microbiological culture result. In incidences of fever (when body temperature was ≥37°C) or suspected infection, the regimen was escalated to a broader spectrum antibiotic (29%, 28/96). In 83.3% (80/96) of cases, patients were prescribed antibiotics throughout their admission with oral antibiotics prescribed at discharge.

### Contextual factors driving infection-related behaviours

The surgical departments in this study were further divided into two independent units, each led by a senior surgeon. There was a hierarchy within the departments, with major decisions taken by the senior surgeons, who referred to their own experience and knowledge in relation to infection management. The level of hierarchy, however, differed between the two units of each surgical department. In both departments, one unit was centrally managed by the unit head, while in the other unit, the hierarchy was less rigid and other members had a say in decision-making.

Both departments had what was considered a small ICU (10 beds in PCVTS and 4 beds in PSx) and the subject of bed availability was a recurring issue during the rounds. Existing patients had to be discharged from the ICU to accommodate new patients being admitted from the operating room (Table 2, Q1).

**Table 2.** Key emerging themes from data collected through observations and interview

Theme	Quote ID	Quote
Contextual factors driving infection-related behaviours	Q1	‘*We operate around 600 to 650 cases per year and we have only a 12-bedded ICU so when you have a critically ill patient who is occupying the ICU and we have four patients being operated, there is definitely lack of beds which is putting pressure on the system*.’—Anaesthetist, PCVTS
	Q2	‘*The surgeon did not want medical patients and surgical patients to be kept together as ‘the flora fauna in both units will be different’. They talked about how the administration was not taking their concerns seriously.*’—Ward round observations, PSx
	Q3	‘*In our department, I know what the organisms are and how we are going to manage and the results are good, but when the patient goes to some other wards, say for example to the cardiac side, then I do not know what organisms are prevalent over there*.’—Surgeon, PSx
	Q4	‘*In multidisciplinary round, everyone crowds around one patient, wants to touch the patient for assessment, which is useless.*’—Anaesthetist, PCVTS
	Q5	‘*I do not know whether the gown really protects the patients as they come in, but I think having these things makes one more conscious about infection overall, so when they wear that gown and come, they become conscious of their actions and even their hand movements also, they will be conscious where they are touching, where they are not, but whether or not it physically acts as a barrier to infection, I am not sure.*’—Cardiology physician
	Q6	‘*They don’t keep new gowns for every use, right. So one carer who is all sweaty, uses the gown. When the other person uses the same gown, I think the gown itself will be a carrier. I don’t feel that wearing a gown is antiseptic or everything, unless the gown is discarded every time. I don’t think wearing the same gown every time that you come is going to help in any way*.’—Surgeon, PSx
Antibiotic prophylaxis extended as a safety net to avert unwanted outcomes	Q7	‘*There is a protocol we follow, for minor surgeries we give a single injection cefotaxime, it will be continued for at least that day, the next day at discharge we routinely give an oral antibiotic for a week… For major surgeries, we follow a triple antibiotic protocol continued for at least one week after the surgery,… after that, we change it to an oral antibiotic. When we discharge the patient we send them on an oral antibiotic usually…*’—*Surgeon, PSx*
	Q8	‘*I have been practising this for the last 10 years and my patients are doing well, why should I change?*’—Surgeon, PSx
Empirical treatment led by the fear of infections	Q9	‘*In a post-cardiac surgery in a newborn, you have to hit hard, so many a time you may have to go up to the higher-end antibiotic*’—*Surgeon, PCVTS*
	Q10	‘*It is always the surgeon’s responsibility. I am sorry to say this, but others are just bystanders. They put their point, but never taken; even if everybody feels that it is wrong prescription, they just cannot pierce through his thinking process*’—Consultant, paediatric cardiology
	Q11	‘*Even though we decide to stop the antibiotic, since the patient is under the surgeon, we have to talk to the surgeon and get his opinion about stopping the antibiotic, so definitely that also depends upon primarily on the surgeon, but definitely we have a say*’—Anaesthetist, PCVTS
Limited role of AMS and IPC teams	Q12	‘*The surgeon absolutely disregards* [AMS policy], *does not bother him, I have indirect knowledge from 2017 and direct knowledge during 2018 that they are unmindful, they are using excessive doses of carbapenem, that it is not rational to combine with an aminoglycoside*’—Anaesthetist, PCVTS
	Q13	‘*I have interacted with juniors in the AMS team. They ask rationale for antibiotics prescribed and it does not go beyond the justification. There is not plenty one-on-one conversation. This is probably because I am lower down in the hierarchy and the conversation happens with seniors.*’—PG fellow, PSx
Multiple players and their influence on antibiotic decision-making	Q14	‘*If I feel that there are a greater number of antibiotics, as junior consultant it is my responsibility to look at the drug charts, the number of days the child has been getting antibiotics, and ask the consultant whether to stop or change it, but the decision rests with us and senior consultant.*’—Surgeon, *PSx*
	Q15	‘*I think we have a tradition of giving more priority to infection control, we have a doctor who is a strong advocate of infection control and he pushes this forcefully. We do our best to the extent possible and we have engaged our nurses. We take care of babies. So whether you are busy or not, you have to do.*’—Paediatric Cardiologist
	Q16	‘*Too many cooks spoil the broth. So, we do the thing. If there is a ventilator patient, we take the help of anaesthetists in ventilator management but as far as antibiotic, resuscitation, IV fluids, TPN, we decide.*’—Surgeon, PSx
	Q17	‘*We ask them (Surgeon), since the patient has been on antibiotic for so many days, is it time to stop or not, but it depends on the patient's condition the doctor will decide.*’—ICU staff nurse

Surgical patients were transferred to medical ICUs or other paediatric or adult wards when needed, to manage the patient flow and bed capacity. This practice was considered an infection risk by the surgeons. (Table 2, Q2 and Q3)

Having a small ICU also meant morning rounds in PCVTS were crowded. Several healthcare workers including surgeons, cardiologists, anaesthetists, residents, nurses, medical social workers and physician assistant, were part of the multidisciplinary ICU rounds, often overcrowding the bed space, risking IPC practices (Table 2, Q4).

The IPC policy differed between the two departments and the location where the patient was admitted. Although the PCVTS department had an IPC protocol in the ICU, it was not consistently applied by the surgeons and paediatric cardiologists, who themselves evaded these rules. Some physicians considered the existence of this rule to be useful for creating a sense of caution among the caregivers regarding the importance of IPC measures in the ICU, whilst having no actual impact on IPC (Table 2, Q5).

The nursing teams in both departments were managed by a single nurse in charge and the ICUs were situated next to each other. Even though the nurses were considered as implementers and gatekeepers of IPC practices, there was a stark difference in their implementation, with fewer or no instructions for carers in PSx (Table 2, Q6).

### Antibiotic prophylaxis extended as a safety net to avert unwanted outcomes

**Figure 1.**
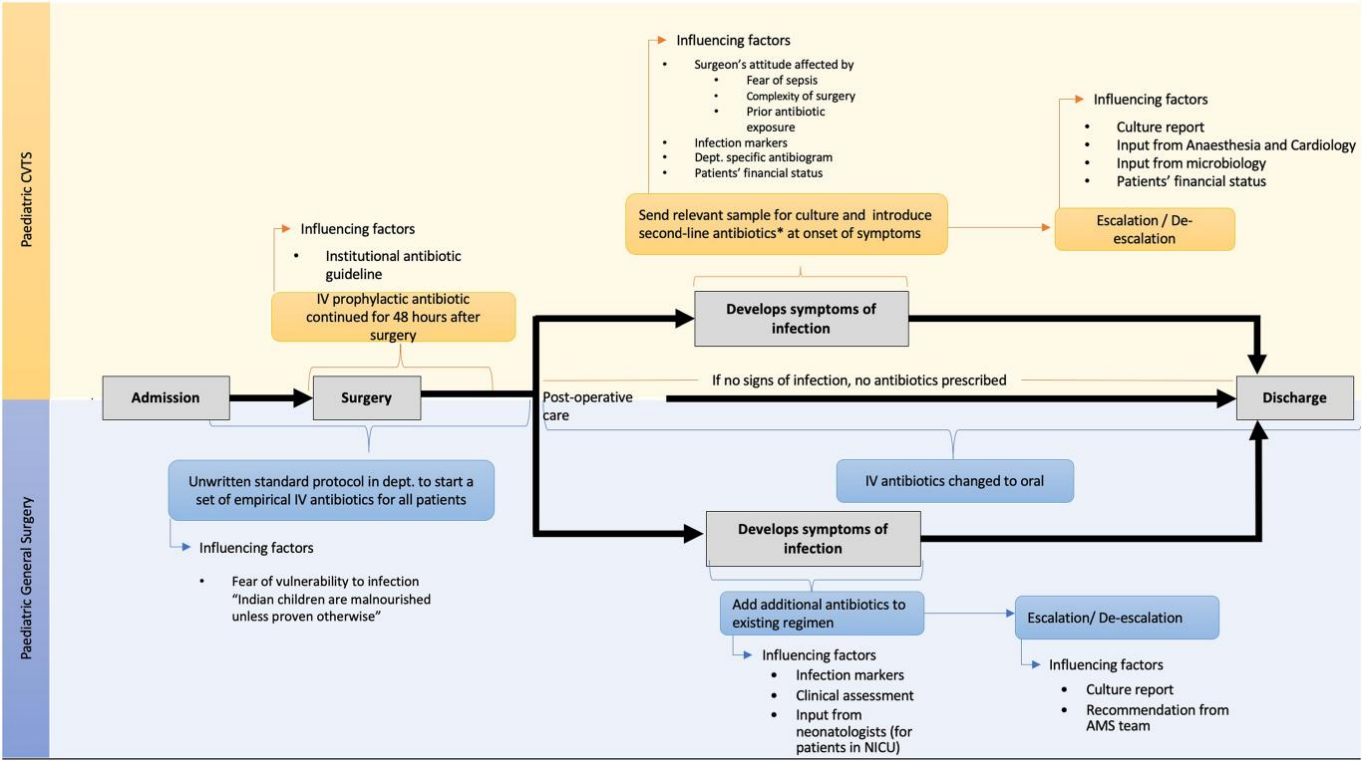
Summary of the typical pathway of both departments and the influencing factors for antibiotic prescribing.

The healthcare workers believed that owing to the high malnutrition rates in India, the antibiotic regimen practised in Western countries cannot be directly adopted into the local context. The PSx surgeons rationalized that ‘all Indian children are malnourished unless proven otherwise’ and because of this, the department had an unwritten policy of giving a predefined set of three antibiotics—cefotaxime, amikacin or ofloxacin and metronidazole—to all patients who were operated on by the team. These antibiotics were continued in IV formulation until the patient could tolerate oral medicines, at which point the regimen was converted to oral formulations, and often continued until or beyond discharge (Table 2, Q7). The influencing factors for antibiotic prescription at each stage in the patient pathway has been summarized in Figure 1.

**Figure 2.**
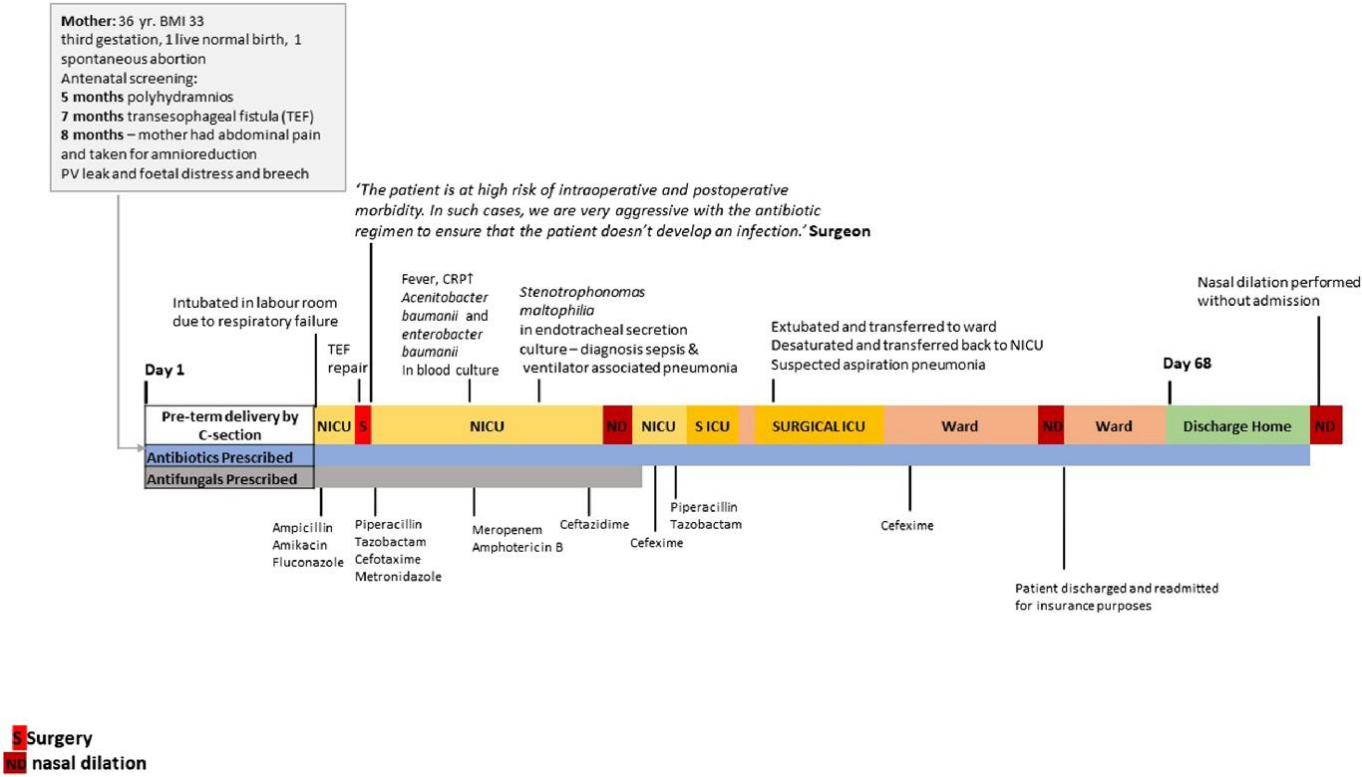
Patient pathway depicting the case study from the paediatric surgery department.

Figure 2 shows a case study of a patient demonstrating the complex pathway and the numerous courses of antibiotics prescribed. It maps the processes for a female neonate who was diagnosed with transoesophageal fistula during the seventh month of gestation. Immediately after delivery, the neonate was admitted to the hospital and underwent corrective surgery. During the 68 day hospital stay and the subsequent period following discharge, the neonate received multiple, overlapping courses of antibiotics. The medical notes did not detail the rationale for these repeated courses of antibiotics. Whilst the prescribers acknowledged that this prolonged antibiotic prophylaxis might not be an optimized approach to manage infections, the practice was justified by the teams by the self-reported 0% SSI rate in the department (Table 2, Q8).

In contrast to the practices of PSx, PCVTS had a more standardized antibiotic prescribing regimen for prophylaxis and empirical antibiotics, which were formed and revised based on recommendations from the IPC team and an antibiogram prepared by the AMS team in the hospital.

### Empirical treatment led by the fear of infections

Being outcome-driven, the doctors often prescribed antibiotics at the earliest indication of infection. There was a reluctance to de-escalate/stop the prescription, even when the infection had resolved or when there were no laboratory results that supported an active infection. This practice was common among the surgeons who, considering themselves responsible for their patients’ health, were averse to changing their practice, referring to consistently low SSI rates in their department as a justification for their behaviour (Table 2, Q9).

The surgeons considered antibiotics in such cases as preventative therapy for patients who may be vulnerable to infection. The team dynamics within each department, differentiated again by each unit and individual attitudes of clinicians, also influenced the way antibiotics were prescribed. The lead surgeon was the primary person responsible for the patient and their clinical outcomes and their decisions are based on years of practice, thereby unquestionable by other team members (Table 2, Q10 and Q11).

### Limited role of AMS and IPC teams

The paediatric surgical departments functioned in a hospital with established IPC and AMS programmes.^22,25^ The influence of such a programme over the departments, however, was limited. They were perceived by the surgical teams to be only a recommending body with limited influence in the decision-making process (Table 2, Q12 and Q13).

The AMS team collaborated with the PCVTS team for co-development of an antibiogram for the department. Though AMS and IPC feedback was generally limited, the surgical teams were interested in sustainable and regular interaction and consultations from the IPC team (Table 2, Q14).

### Multiple players and their influence on antibiotic decision-making

Whilst the established policy in PSx was to prescribe antibiotics irrespective of infection, a few team members were not convinced of the rationale but did not consider it necessary to change the policy due to their unit’s low reported SSI rates (Table 2, Q14).

The PCVTS team was more multidisciplinary, and there were more actors with a role in AMS and IPC. Anaesthetists and cardiologists were available round the clock and were part of the daily rounds and they contributed to the antibiotic decision-making for patients (Table 2, Q15).

In contrast to the PCVTS department, involvement of the anaesthesia team in routine patient care was not seen to be a requirement by the PSx team. In the case of an anaesthetist being required for a patient, they sought assistance from the adult gastrointestinal anaesthesia team. The surgeons also had a good rapport with the neonatologists who were involved in managing their patients in neonatal ICU (NICU) (Table 2, Q16).

Despite the multidisciplinary teams in both the departments, it was the surgeon who made the final call regarding patient management and the other team members complied irrespective of them being on board with the plan or not (Table 2, Q17).

## Discussion

In this study, we explored the behaviours and practices that impacted the antibiotic decision-making in two paediatric surgical departments in a tertiary teaching hospital in Kerala, India. There have been studies with similar findings regarding the influence of the attitudes and leadership style of the surgical team on antibiotic decision-making.^26^ Within paediatric specialties, other factors, such as the perception of vulnerability of the patients, are brought into the decision-making. Whilst there is a high-functioning multidisciplinary team in paediatric surgery, we found that the surgeon’s decision was respected, even when there were conflicting points of view.

The contribution of anaesthetists and cardiologists in patient care can be overlooked and overruled by the surgeons taking the lead role, causing dissatisfaction and tension among other team members. The fear of negative infection-related outcomes and the complexities of dealing with what they consider vulnerable paediatric patients puts them on high alert for using antibiotic prescribing as a safety net. This means the perceived short-term gain of prescribing antibiotics outweighs the longer-term consequences of AMR, from the surgeons’ perspective. This practice is further rationalized by the prescribers by reference to their surgical outcome data. The longer-term consequences of unnecessary prolonged antibiotic prophylaxis, and escalation to broad-spectrum antibiotics when infection markers are raised are not given consideration. In addition, inappropriate swabbing of wounds could lead to positive culture results that do not necessarily represent a clinical infection.^27^ This misinterpretation can subsequently drive unnecessary antibiotic prescribing, contributing to AMR and adverse patient outcomes.

The current literature on AMS in paediatric populations focuses on the role and impact of AMS programmes and does not factor in surgical specialty drivers of behaviours, which may be in direct conflict with AMS objectives. Our study has shown that surgeons’ and the other associated team members’ behaviour and practices have an influence on the overall IPC and AMS practices. AMS strategies in surgical specialties need to be contextualized to the normative practices and risk perception in surgical teams. This calls for the need to tailor the language of risk around antibiotic use to different specialties and use of data that will be of relevance to the different specialties, particularly in patient populations considered at high risk of acquiring infection, such as in the NICU.

Surgeons have the ultimate responsibility for the outcomes in their patient populations. While IPC and AMS teams have been set up to manage emergence and spread of AMR and the effective management of infections,^5,28,29^ their functions are not fully integrated into the medical and surgical specialties. Engaging with the surgical specialties becomes even more difficult where the decision-making is outcome-driven. There remain communication gaps between medical, AMS and IPC, and surgical teams, which is a barrier to effective decision-making. This was also identified in a similar study in the adult surgical pathway, where the IPC and AMS teams remain more of a consulting service than active contributors to the care of the surgical teams.^2^ While the composition of the IPC and AMS teams varies across different settings, emerging studies from LMICs support multidisciplinary AMS teams in general, as well as being specific to paediatric departments.^5,28,30–33^ Our findings highlight this need, in addition to implementation of tailored interventions that identify the prioritization of outcome-driven risk in the NICU.

### Limitation

The study was conducted in a hospital that is known for implementing advanced interventions and policies related to infection prevention and AMS, which is atypical of the local or national context. The setting also has uncommon characteristics that differ from other international settings.^34,35^ Therefore, the findings may not be directly applicable to different contexts or settings.

### Conclusions

Surgical decision-making is outcome-driven. In infection management, this leads to overprescription of empirical antibiotics and unnecessary prolongation of surgical prophylaxis. The rationale for such inappropriate use in this study was complicated by the surgeons’ beliefs about the contextual determinants of health in India. To optimize infection-related practices in surgical teams it is critical to engage with the senior clinicians about their risk perception in relation to infections in the NICU and how they can be effectively managed through AMS.

## Supplementary Material

dlae105_Supplementary_Data

## Supplementary data

File S1 is available as Supplementary data at *JAC-AMR* Online.
