# Supplementary material for: Rationalizing irrational prescribing—infection-related attitudes and practices across paediatric surgery specialties in a hospital in South India
Source: JAC Antimicrob Resist. 2024 Jul 13;6(4):dlae105. doi: 10.1093/jacamr/dlae105 (PMC11245696; doi:10.1093/jacamr/dlae105)
Supplement: dlae105_Supplementary_Data [file dlae105_supplementary_data.docx]

| **Demographics** |  | | | | | |
| --- | --- | --- | --- | --- | --- | --- |
| Patient identification number: |  | | | | | |
| Age/Sex: | Location: | | | | | |
| Department: | Admitting physician: | | | | | |
| Date of Admission : | Date of Review: | | | | | |
| Date of Discharge: | Duration of hospital stay: | | | | | |
| Diagnosis : |  | | | | | |
| **Surgical Procedure data** |  | | | | | |
| Surgery | Elective or emergency | | | | | |
| Type of operation |  | | | | | |
| Date of operation |  | | | | | |
| Antibiotic prophylaxis given |  | | | | | |
| If yes, antibiotic details | Name | | | | dose | |
|  | frequency | | | |  | |
| **Cultures** |  | | |  | | |
|  | Culture 1 | | | | Culture 2 | |
| Date |  | | | |  | |
| Specimen |  | | | |  | |
| Organism |  | | | |  | |
| Antibiotic sensitivity |  | | | |  | |
| **Infection markers on day of initiating antibiotics** | 48 hours prior to starting antibiotics | | | | 24 hours prior to starting antibiotics | |
| Temperature(^0^F) |  | | | |  | |
| WBC(K/uL) |  | | | |  | |
| CRP(mg/L) |  | | | |  | |
| Procalcitonin |  | | | |  | |
| **Chest X – ray** |  | | | | | |
| Date: |  | | | | | |
| Report |  | | | | | |
| **Post operative antibiotic** |  | | | | | |
|  | Antibiotic 1 | Antibiotic 2 | Antibiotic 3 | | | Antibiotic 4 |
| Indication |  |  |  | | |  |
| Name |  |  |  | | |  |
| Route |  |  |  | | |  |
| Dose |  |  |  | | |  |
| Frequency |  |  |  | | |  |
| Start date |  |  |  | | |  |
| End date |  |  |  | | |  |
| Duration of therapy |  |  |  | | |  |
| Compliance to local policy |  |  |  | | |  |
| **30 day follow up(YES/NO)** |  | | | | | |
| Readmission |  | | | | | |
| Mortality |  | | | | | |

Data definitions

| Patient identification number: | Unique patient number given by the hospital/ project |
| --- | --- |
| Age/Sex: | Age in years; Sex to be either entered as male or female |
| Department and Admitting physician: | Surgical department and the name of the physician under whom patient is admitted |
| Date of Review: | Date on which the case is collected |
| Duration of hospital stay: | Number of days patient was admitted in the hospital |
| Diagnosis : | Surgical diagnosis to be entered |
| Surgery | Elective: surgery that is scheduled in advance |
|  | Emergency: immediate surgery due to a medical emergency |
| Type of operation | Name of the surgery that was performed |
| Antibiotic prophylaxis given | Administration of an antibiotic prior to surgery to prevent surgical infection post procedure |
| If yes, antibiotic details | Name of the antibiotic as entered in the patient's file |
|  | Dose: Amount of the antibiotic administered (mg , g, MU ) |
| Specimen of culture | The sample that was send for culture(blood/urine/sputum/BAL/tissue) |
| Organism | The name of the bacteria isolated in the culture as per the microbiology report from hospital information system |
| Antibiotic sensitivity | Names of the antibiotics that are shown to be sensitive to the isolated bacteria as per the microbiology report from hospital information system |
| Temperature(^0^F) | Temperature above 98.6^0^F at 24 and 48 hours prior to initiation of antibiotic to be noted |
| WBC(K/uL) | Count at 24 and 48 hours prior to initiation of antibiotic to be noted (Reference range- 4-10K/uL) |
| CRP(mg/L) | Count at 24 and 48 hours prior to initiation of antibiotic to be noted (Reference range-0-10mg/L) |
| Procalcitonin | Count prior to starting of antibiotic (Reference range- 0-0.046ng/mL) |
| Chest X – ray | Presence of localized or patchy or diffuse infiltrate in chest x-ray is suggestive of infection. |
| Post operative antibiotic | antibiotic initiated .other than the prophylaxis, to treat an infection |
| Indication | the reason for which the antibiotic was initiated |
| Name | Name of the antibiotic as entered in the patient's file |
| Route | route by which the antibiotic has been administered (IV/PO) |
| Dose | amount of the antibiotic that has been prescribed |
| Frequency | time interval at which drug is administered (OD/BD/TID/QID/STAT) |
| Start date | date of initiating antibiotic |
| End date | date of stopping the antibiotic |
| Duration of therapy | number of days for which the patient received antibiotic |
| Compliance to local policy | whether the antibiotic prescribed was according to the hospital antibiotic policy/ antibiogram |
| 30 day follow up | to follow up the patient for 30 days from the date of discharge |
| Readmission | to note if the patient was admitted anytime during this 30day time period |
| Mortality | mortality at the end of 30 day time period |
